# Pupille d'Adie isolée: à propos d'un cas

**DOI:** 10.11604/pamj.2015.20.330.4437

**Published:** 2015-04-07

**Authors:** Zineb Jaja, Mina Laghmari, Omar Lazrek, Rajae Daoudi

**Affiliations:** 1 Université Mohamed V, Hôpital des Spécialités, Rabat, Maroc

**Keywords:** pupille, adie, hépatite auto-immune

## Abstract

La pupille d'adie correspond à une mydriase unilatérale qui ne repond pas à la stimulation lumineuse et persiste à la vision de prés. La pupille d'adie est généralement associée à de nombreuses pathologies comme la maladie cœ**l**ique, l'hépatite auto-immune, la migraine. Nous rapportons le cas d'une jeune fille présentant depuis 2 ans une pupille d'adie isolée. La pupille d'adie est une manifestation rare, l'atteinte isolée chez notre patiente ne permet pas de mettre un diagnostic précis à son atteinte.

## Introduction

**Figure 1:**
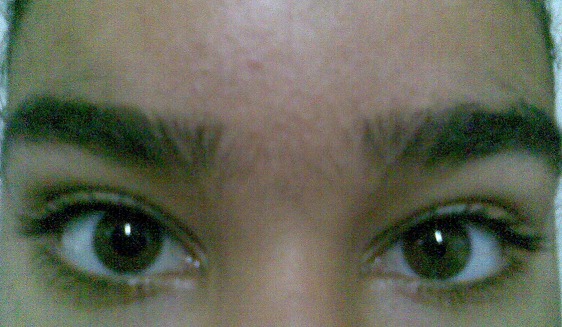
Pupille d'adie (œil droit) qui est resté dilaté à l’éclairage

La pupille d'adie correspond à une mydriase unilatérale qui ne repond pas à la stimulation lumineuse et persisite à la vision de prés. Elle est plus fréquente chez les femmes [1], son diagnostic est obtenu grâce au test à la pilo diluée à 0,125%. La pupille d'adie est generalement associée à de nombreuses pathologies comme la maladie cœlique, l'hépatite auto-immune, a migraine [2, 3]. Ou peut ête incluse dans le cadre de certains syndromes comme le syndrome d'adie-Holmes caractérisé par son association avec une abolition des reflexes ostéo-tendineux. Nous rapportons le cas d'une jeune fille présentant depuis 2 ans une pupille d'adie isolée (Figure 1).

## Patient et observation

**Figure 2:**
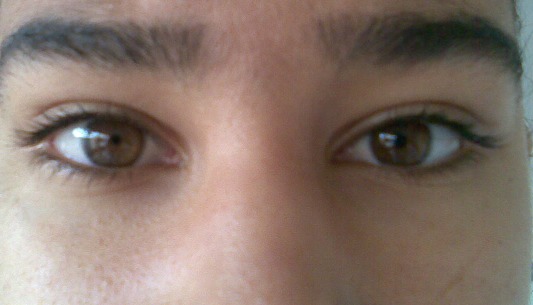
Pupille en myosis après le test à la pilocarpine confirmant le diagnostic de la pupille d'adie

Il s'agit d'une jeune fille âgée de 17 ans, sans antecedants particuliers, ayant constatée d'elle même une anisocorie sans signes accompagnateurs. A l'examen on trouve une AV à 8/10 OF ameliorable à 10/10 après une corrrection par + 1, une AV à 10/10 OG. A l'eclairage on constate que la pupille de l'OD reste dilatée alors que la pupille de l'OG se met en myosis, on constate le même chose à la vision de pres (Figure 1). Le fond d’œil est normal en ODG. Le test à la pilo diluée à 0.125 est positif (Figure 2); on conclue qu'il s'agit d'une pupille d'adie.

## Discussion

La pupille d'adie est une manifestation rare, caractérisée par une pupille dilatée légèrement réactive ou irréactive à la stimulation par la lumière. Cette atteinte est en rapport avec une dénervation du segment post ganglionnaire du sphincter pupillaire et du muscle ciliaire. Cette atteinte est unilatérale dans 80% des cas [4, 5]. Quand elle est associée à une diminution ou absence de réflexes osteotendineux elle est incluse dans le syndrome d'adie -Holemes. A l'examen la pupille est large et régulière avec absence de réponse à l'exposition à la lumière ceci est associé à des mouvements vermiformes des bords de la pupille. Le diagnostic esr confirmé au test à la pilo diluée à 0.125%. La pilocarpine diluée donne une constriction car l'effet de la pilocarpine diluée est augmenté quand il ya une dénervation parasympathique. Ce test ne permet non seulement de faire le diagnostic positif mais aussi de différencier entre pupille tonique arréactive et une lésion nerveuse pré-ganglionnaire.

## Conclusion

La pupille d'adie est une manifestation rare, l'atteinte isolée chez notre patiente ne permet pas de mettre un diagnostic précis à son atteinte.
